# *gyrA* Mutations in Fluoroquinolone-resistant *Clostridium difficile* PCR-027

**DOI:** 10.3201/eid1303.060771

**Published:** 2007-03

**Authors:** Denise Drudy, Lorraine Kyne, Rebecca O’Mahony, Séamus Fanning

**Affiliations:** * University College Dublin, Dublin, Ireland; † Mater Misericordiae University Hospital, Dublin, Ireland

**Keywords:** Hypervirulent Clostridium difficile, fluoroquinolone resistance, gyrA mutations, letter

**To the Editor:** *Clostridium difficile* is the most common cause of bacterial diarrhea in hospitalized patients (*1*). Antimicrobial drug therapy is the most important risk factor associated with the acquistion of *C. difficile,* and several antimicrobial agents including clindamycin, amoxicillin, and cephalosporins have been particularly associated with *C. difficile* infection (*2*). Acquisition of resistance to clindamycin is considered 1 mechanism whereby clonal strains emerge and predominate in healthcare environments (*3*). Historically, fluoroquinolone antimicrobial agents were considered low risk for *C. difficile–*associated-disease; however, recent studies indicate a shift in the risk associated with their use (*4*). Furthermore, recent outbreaks in Canada and the United States have been associated with fluoroquinolone exposure (*4*).

Recently, several *C. difficile* outbreaks due to PCR ribotype 027 (PCR-027) and associated with increased disease severity and death have been reported worldwide (*4*). This strain type contains the genes for binary toxin and has an 18-bp deletion and a frameshift mutation in *tcdC* hypothesized to result in deregulated expression of toxins A and B. These strains produce 16× more toxin A and 23× more toxin B in vitro than toxinotype 0 strains (*5*). These isolates demonstrate universal high-level resistance to fluoroquinolones in contrast to that of PCR 027 isolates collected before 2001 (*4*).

We report the mechanism of fluoroquinolone resistance in a cluster (n = 5) of Irish PCR-027 *C. difficile* isolates that were characterized by using toxinotyping and 16–23S ribotyping. Amplification with PCR and sequencing was used to identify the binary toxin gene (*cdtB*) and an 18-bp deletion and a frameshift mutation at position 117 in the *tcdC* gene. Antimicrobial susceptibility to 5 fluoroquinolone antimicrobial drugs was determined with E-tests (AB-Biodisk, Solna, Sweden). The quinolone-resistance–determining region (QRDR) of *gyrA* and *gyrB* was amplified by PCR and characterized. The nucleotide sequence data for partial sequences of the *gyrA* gene were submitted to GenBank and assigned accession nos. DQ821481, DQ821482, DQ821483, and DQ821484.

PCR ribotyping profiles identified 1 cluster of *C. difficile* PCR-027 with clinical isolates that showed indistinguishable profiles to the control 027 strain. PCR identified the *cdtB,* an 18-bp deletion, and a frameshift mutation at position 117 in the *tcdC* gene in all 5 isolates. These strains were universally resistant to the fluoroquinolones tested (ofloxacin, ciprofloxacin, levofloxacin, moxifloxacin, and gatifloxacin, respectively, MIC >32 μg/mL [Table]). Control isolates were susceptible to moxifloxacin and gatifloxacin (MICs 0.3, 0.2 μg/mL, respectively); however, these strains had reduced susceptibility to levofloxacin (MIC 3 μg/mL) and were resistant to ciprofloxacin and ofloxacin (Table). Sequence analysis determined that all 5 PCR-027 isolates had a single transition mutation (C to T), resulting in the amino acid substitution Thr-82-Ile in *gyrA* (Table). No amino acid substitutions were found in the QRDR of *gyrB* (data not shown).

**Table:** Characterization of representative isolates, Ireland, 2006

Isolate	Toxigenic status	Ribotype	Fluoroquinolone MIC μg/mL					Amino acid substitution
			Ciprofloxacin	Ofloxacin	Levofloxacin	Gatifloxacin	Moxifloxacin	
1470*	A^-^B^+^	017	>32	>32	3	0.38	0.25	Thr 82
VPI10463*	A^+^B^+^	D	>32	>32	3	0.38	0.25	Thr 82
CD 196*	A^+^B^+^	027	>32	>32	3	0.38	0.25	Thr 82
M216†	A^+^B^+^	027	>32	>32	>32	>32	>32	Thr-82-Iso
C2191†	A^+^B^+^	027	>32	>32	>32	>32	>32	Thr-82-Iso
V6-13†	A^+^B^+^	027	>32	>32	>32	>32	>32	Thr-82-Iso
V6-15†	A^+^B^+^	027	>32	>32	>32	>32	>32	Thr-82-Iso
V6-20†	A^+^B^+^	027	>32	>32	>32	>32	>3	Thr-82-Iso

*Control isolates VPI-10463, 1470 CD196. †Clinical 027 isolates from 3 different institutions investigated in this study.

Mutations in the active site or the QRDR of DNA gyrase and topoisomerase IV have been associated with increased resistance to fluoroquinolones in several bacteria (*6*). This report identifies for the first time a mutation in *gyrA* that is associated with high-level resistance to fluoroquinolones in *C. difficile* PCR-027. In *Escherichia coli*, amino acid substitutions that occur at Ser-83 in *gyrA* have been associated with fluoroquinolone resistance (*6*). Thr-82 in *C. difficile* corresponds to Ser-83 in *E. coli*. Thr-to-Ile amino acid substitutions corresponding to Ser-83 have been associated with fluoroquinolone resistance in several bacteria, including *Pseudomonas aeruginosa*, *Enterobacter aerogenes*, *Campylobacter jejuni,* and *C. difficile* (*6*). Ackermann et al. described 2 mutations in *gyrA* that resulted in an amino acid substitution corresponding to codon 83 in *E. coli*. Thirteen of the 18 *C. difficile* isolates had the Thr-82-Ile substitution, and 1 strain had a Thr-82-Val substitution (*7*). Dridi et al. described this Thr-82-Ile GyrA substitution in 6 resistant *C. difficile* strains corresponding to 3 serogroups, H1, A9, and 1C (*8*).

Early studies investigating fluoroquinolone antimicrobial agents suggested that most *C. difficile* isolates were susceptible to these drugs. Antimicrobial drug resistance to this class has increased with fluoroquinolone use, and currently these drugs remain the most frequently prescribed antimicrobial agents in the United States and Europe. Acquired resistance to the newer fluoroquinolone antimicrobial agents is not restricted to ribotype PCR-027, although different amino acid substitutions in the QRDR of *gyrA* and *gyrB* have been described (*7*–*9*). Wilcox et al. have described high-level fluoroquinolone resistance in PCR ribotype-001, an endemic strain type found in several healthcare settings in the United Kingdom (*10*). We have previously described the emergence of a fluoroquinolone-resistant toxin A–, toxin B–positive strain in Dublin (*9*).

We report a mutation in *gyrA* associated with fluoroquinolone resistance in *C. difficile* PCR-027. Antimicrobial drug resistance in *C. difficile* isolates must be monitored because the emergence of universal fluoroquinolone resistance in different *C. difficile* strain types may be a factor promoting outbreaks in hospitals. As exposure to several different fluoroquinolone antimicrobial drugs have been independently associated with *C. difficile–*associated-disease, restricted use of all fluoroquinolones, rather than changing from 1 quinolone to another, may be a necessary step toward preventing and controlling *C. difficile* outbreaks.
